# Ergonomic Challenges Inherent in Neonatal Resuscitation

**DOI:** 10.3390/children6060074

**Published:** 2019-06-03

**Authors:** Nicole K. Yamada, Janene H. Fuerch, Louis P. Halamek

**Affiliations:** 1 Division of Neonatal and Developmental Medicine, Department of Pediatrics, Stanford University, Palo Alto, CA 94304, USA; jfuerch@stanford.edu (J.H.F.); halamek@stanford.edu (L.P.H.); 2 Center for Advanced Pediatric and Perinatal Education (CAPE), Stanford University, Palo Alto, CA 94304, USA

**Keywords:** neonatal resuscitation, human performance, human factors and ergonomics, physical ergonomics, macroergonomics, cognitive ergonomics

## Abstract

Neonatal resuscitation demands that healthcare professionals perform cognitive and technical tasks while working under time pressure as a team in order to provide efficient and effective care. Neonatal resuscitation teams simultaneously process and act upon multiple data streams, perform ergonomically challenging technical procedures, and coordinate their actions within a small physical space. An understanding and application of human factors and ergonomics science broadens the areas of need in resuscitation research, and will lead to enhanced technologies, systems, and work environments that support human limitations and maximize human performance during neonatal resuscitation.

## 1. Introduction

At birth, newborn infants must transition from intrauterine to extrauterine physiology in order to sustain life via pulmonary respiration rather than relying on placental gas exchange. Approximately 85% of newborns born at term will initiate spontaneous breathing within 10 to 30 s of birth [1]. Conversely, approximately 10% of all newborns will require drying and stimulation to begin breathing after birth and another 3% will initiate respiration after positive pressure ventilation [1,2]. The science and recommendations on neonatal resuscitation have evolved to optimize the physiological support provided by healthcare professionals (HCPs) to newborn infants in distress, albeit with minimal consideration of the challenges faced by the HCPs tasked with implementing these interventions. Issues such as the physical space necessary to accommodate a neonatal resuscitation team, objective ways to measure the technical performance of invasive procedures, and the human capacity for processing multiple data streams are important considerations to facilitate safe, effective, and efficient delivery of care to neonates in need of support after birth.

Human factors and ergonomics is a discipline that can address this gap. Experts in this field specifically study the interaction of humans with the elements of their environment, and how each affects the performance of the other. The goals of human factors practitioners are to optimize the performance of the system as a whole (human plus environment), increase safety, and improve user satisfaction [3]. The field draws from multiple disciplines, including but not limited to cognitive science, psychology, industrial engineering, mechanical engineering, biomechanics, anthropometry, and physiology.

Within the field of human factors and ergonomics, there are three major areas of study: (a) physical, (b) organizational, and (c) cognitive ergonomics [4]. Physical ergonomics refers to those elements related to human anthropometry, biomechanics, and physiology that affect the human ability to physically interact with the environment and perform tasks. Organizational ergonomics—often referred to as “macroergonomics”—is the study of interactions among groups of people and their work environment, organizational structures, and policies. Topics studied in organizational ergonomics include communication, teamwork, and quality management. Finally, cognitive ergonomics relates to aspects of human mental processes and function, such as vision, attention, memory, decision-making, and problem solving, and how they affect human ability to perceive and understand the environment. This manuscript will use specific examples from neonatal resuscitation to demonstrate how knowledge of physical, organizational, and cognitive ergonomics can inform training, organizational structure, and device and environment design to optimize human and team performance in healthcare.

## 2. Physical Ergonomic Challenges

A number of physical ergonomic challenges and knowledge gaps exist in the field of neonatal resuscitation. Some of these include: (a) a paucity of objective data to define the optimal performance of invasive procedures, (b) the wide anthropometric variation of HCPs on resuscitation teams, and (c) inadequate clinical space to accommodate neonatal resuscitation teams in the intrapartum environments of many hospitals.

### 2.1. Objective Measurement of the Forces of Endotracheal Intubation

Endotracheal intubation is a life-saving procedure that is performed during neonatal resuscitation in the setting of apnea, respiratory insufficiency, or bradycardia that is unresponsive to positive-pressure ventilation by non-invasive ventilation methods such as face mask ventilation [2,5]. Despite the frequency with which intubation is performed in urgent or emergent circumstances in the delivery room and other healthcare environments, there is little objective data that can be used to distinguish between optimal and suboptimal performance of this procedure. HCPs with minimal experience in intubation learn the procedure through teaching and coaching from instructors who provide guidance to the learner based on feedback via shared view of a videolaryngoscope at best, and via verbal description of the learner’s visualization of the airway at worst. Intubation success is directly correlated with experience level; studies have shown that only 20%–50% of infants are intubated on the first attempt, and up to 40% require three or more attempts [6,7,8,9,10,11,12]. Improper intubation technique in any patient can lead to life-threatening injuries such as soft tissue lacerations and tracheal perforation. Failed, lengthy, or multiple intubation attempts can lead to prolonged duration of the adverse physiological changes associated with intubation, including increased intracranial pressure, bradycardia, and hypoxemia, as well as increased incidence of adverse events such as esophageal intubation and airway trauma [13,14,15,16,17,18].

The use of a laryngoscope to open the mouth and expose the glottic structures has components that can be further analyzed objectively using knowledge of physical ergonomics. For example, the degree of force that can be safely applied to the soft tissues such as the tongue and posterior oropharynx and the degree of torque used to manipulate the airway in order to expose the trachea with the laryngoscope are essentially unknown. While a number of investigators have begun to study these factors in simulated and real adult patients [19,20,21,22,23,24,25,26,27,28,29], this data cannot be directly translated to neonatal patients due to different equipment used and the large differences in patient weight and size. Furthermore, the immature and more delicate tissues of newborns are at greater risk of trauma, and their anterior tracheal anatomy can make intubation more challenging than in older patients. In the absence of data that accurately describe the force and torque that is safely used by experts during neonatal intubation and the lack of devices capable of measuring those variables in real time, there is a great need for objective data to guide practicing HCPs who deliver neonatal care or novices undergoing training in correct technique while simultaneously minimizing the risk of patient injury.

### 2.2. Biomechanics during the Performance of Resuscitation Procedures

Physical ergonomics can also be applied to objectively measure biomechanical challenges faced by HCPs while delivering patient care. The anthropometric variation of the population of HCPs who comprise neonatal resuscitation teams—and, in fact, nearly all healthcare teams—is not currently considered when designing devices or environments used for resuscitation. Understandably, individuals are selected for resuscitation teams based on their skills in the tasks necessary to deliver emergency care to neonates in distress. While the ability to deliver high-quality patient care is indeed the first priority, each individual’s biomechanics while performing resuscitation procedures cannot be ignored. Any given team of HCPs may span the range of the population anthropometry and the “optimal” posture, position, and technique for an individual of one combination of height, weight, and various limb lengths is unlikely to be sufficient for the other individuals on the same team.

In the context of a life-saving but physically fatiguing intervention such as chest compressions, knowledge of optimal physical ergonomics is particularly useful in order to maximize the potential of HCPs of any anthropometry to effectively administer that intervention for the duration of a resuscitation. There has been little investigation in this area within the field of neonatal resuscitation. Investigators in adult cardiopulmonary resuscitation and emergency medicine have examined the ergonomics of chest compressions in adult patients from the perspective of HCP fatigue, quality of chest compressions over time, and with the patient lying on different bed surfaces. For example, several studies have compared the quality of chest compressions delivered with the patient lying on the floor to those delivered with the patient on a stretcher or bed [30,31,32]. A study of bed height found that mean chest compression depth in adult resuscitation was significantly lower at higher bed heights [33]. Other investigators have studied the quality of chest compressions delivered with the HCP positioned at varying lateral distances from the adult patient’s chest, due to either the posture selected by the HCP or the width of the bed on which the patient was lying [34,35]. In these studies, chest compressions were more effective when the HCP was positioned in closest proximity to the patient. While these studies inform how HCP positioning and posture may affect the quality of chest compressions in adult patients, only one study has specifically evaluated chest compressions performed during neonatal resuscitation [30]. Additional research on the optimal ergonomics and metrics to assess the quality of chest compressions during neonatal resuscitation is sorely needed.

The study of the physical ergonomics of technical procedures during resuscitation can also lead to findings that may drive future device design that better meets the needs of all users. For example, in a biomechanical analysis of HCPs during neonatal resuscitation procedures, the preferred bed height for the performance of endotracheal intubation was 14 centimeters (cm) higher (*p* < 0.05) than the preferred bed height for the performance of chest compressions [36]. However, the foot pedal that allows adjustment of the bed height on many infant resuscitation beds is readily accessible only to those HCPs standing on the sides of the bed. The HCP at the infant’s head manages the patient’s airway and is responsible for endotracheal intubation, but this person is positioned farthest from the mechanism used to adjust the bed height. Additionally, there is no ability to adjust the lateral angle or width of the bed in order to accommodate the variable stature, horizontal reach, and abdominal depth of the HCPs on the resuscitation team. As a result, HCPs make a number of postural adjustments to perform life-saving interventions such intubation and chest compressions. During chest compressions, for example, HCPs lean over the center of the bed and rotate the torso and spine in order to reach the infant and correctly position their hands around the infant’s thorax. These non-neutral postures are potential risk factors for HCP injury as well as unsuccessful or inappropriate procedure performance. Studies of the physical ergonomics of HCPs performing resuscitation have the potential to improve the safety of those interventions, the efficacy with which they are executed, and to better understand the optimal design of the medical devices used.

### 2.3. Physical Space Constraints for Neonatal Resuscitation

Regardless of the age or size of the patient, the acuity of resuscitation necessitates that large teams of HCPs gather around the bed of a single patient. In neonatal resuscitation, this team construct highlights the physical limitations of the clinical workspace. The most premature neonates weigh as little as 400–500 g and measure merely 30 cm in length. The bed surface on which the newly born infant is placed is approximately 65 cm × 50 cm in area. Yet, between two and four adult HCPs may require simultaneous access to the infant to perform various tasks during neonatal resuscitation.

Beyond the limitations of the space around the patient’s bed, neonatal resuscitation is further impacted in many hospitals by the lack of sufficient physical space allocated to neonatal care in the typical labor and delivery or operative delivery rooms. Historically, hospitals and the patient bed space were not designed with the specific goal of enhancing staff and patient safety [37]. In many institutions, the design of and space allocated to labor and delivery wards is instead heavily influenced by a combination of building codes, budget constraints, and maternal patient experience and satisfaction. Because normal labor, delivery, and neonatal transition are the more common occurrences, anticipating and planning for intrapartum obstetrical and neonatal emergencies is often an afterthought during design. However, increasing acuity of maternal or neonatal care correlates with an increase in the number of HCPs necessary for care. These physical space requirements can prove inadequate when full maternal and neonatal resuscitation teams assemble in a delivery room. The impact of physical design on maternal clinical outcomes and patient safety is starting to be recognized, but the effect of space constraints on the performance of neonatal resuscitation teams and patient outcomes is yet to be considered [38,39,40]. As a result, the ideal physical design to optimize human performance is unknown at this time.

## 3. Macroergonomic Challenges

Resuscitation teams throughout healthcare are multidisciplinary by definition, and the members of these teams are not typically co-located at the time of their activation. The assembly of resuscitation teams routinely requires the smooth execution of policies and processes and the seamless functioning of communication systems. In neonatal resuscitation, common macroergonomic challenges include summoning the neonatal resuscitation team to the delivery room and the communication of vital information between and within multidisciplinary teams.

### 3.1. Summoning the Neonatal Resuscitation Team

The call for the neonatal resuscitation team is an opportunity to relay key information that is vital to ensuring that the team has the right information at the right time and is adequately prepared to resuscitate and stabilize the newborn infant [41,42]. However, there can be significant practice variation between and even within centers regarding how the neonatal resuscitation team is activated. For example, prior to the implementation of a quality improvement effort aimed at improving this communication, one center identified that only 8% of pages to the neonatal resuscitation team contained the fetus’ gestational age and just 33% of pages contained the reason for requesting the neonatal resuscitation team [43]. The process that resulted from this quality improvement work included a number of systems-based strategies to improve communication between the obstetric team and the neonatal resuscitation team, including the training of staff, a decrease in the number of categories of information that were transmitted, and the implementation of a software-based program to aid standardized information gathering and dissemination [43].

An understanding of ergonomics and human behavior is critical to finding and applying operational solutions to macroergonomic problems such as communication between healthcare teams. This was demonstrated by the same authors following a change in the communication system used to summon the neonatal resuscitation team; the implementation of a call center responsible for paging all emergency response teams in the entire hospital resulted in significant and unexpected negative effects. Mandating that phone calls to summon the neonatal resuscitation team be routed through this call center led to a substantial increase in the inappropriate use of a code button by labor and delivery staff, who preferred to circumvent the call center altogether [42]. Communicating with the call center staff was seen as less timely and more cumbersome than previous methods of summoning the neonatal resuscitation team and the swift response that could be obtained by pressing the code button. In addition, the ensuing arrival of HCPs who were not needed at a neonatal resuscitation but who responded as members of the code team added confusion to the situation. While seen as a relatively small system change for the hospital, the change in process to using the call center caused widespread staff dissatisfaction and led to behaviors that could have adversely impacted patient care.

### 3.2. Communication between Teams and within Teams

Following the arrival of the neonatal resuscitation team to a newborn delivery, the interaction between the obstetric and pediatric teams is an inherent macroergonomic challenge of neonatal resuscitation. Antepartum and intrapartum risk factors can have a direct effect on the risk for and need for neonatal resuscitation, and the relevant information must be communicated in a timely fashion between obstetric and neonatal teams [5,44]. Coordination of care and communication with the neonatal care team even outside of resuscitation has been identified as a common challenge for labor and delivery staff [45]. Communication in the labor and delivery environment during and immediately after childbirth is like no other environment in healthcare, as it requires navigation of the simultaneous care of two (or more) patients by multiple teams within a single room. As many as 10–12 HCPs from at least three different medical teams (obstetrics, anesthesia, and pediatrics) and five disciplines (physicians, nurses, respiratory care practitioners, advanced practice professionals, and surgical technicians) may convene and must coordinate their care to ensure the safety of the mother and neonate(s) within a short period of time [46]. In addition, different members of each team may arrive into the room at varying times (i.e., rather than as an intact team entering at a single timepoint), leading to the need to repeat information that has already been communicated or have some team members remain unaware of important pieces of information that could affect decision-making about subsequent patient care. In one study of communication among labor and delivery nurses, technicians, obstetricians, and anesthesiologists during simulated labor and delivery emergencies, up to 30% of questions between HCPs were repeated in each scenario [47]. Communication has also been identified as a macroergonomic challenge for neonatal resuscitation teams, with poor communication being correlated with poor adherence to the Neonatal Resuscitation Program (NRP) algorithm [48].

While a number of standardized communication tools have been developed in healthcare to facilitate the sharing of information, these are primarily designed for communication between HCPs during the transfer of care or updates on status of care between HCPs [49,50,51,52]. These tools are limited in their applicability to emergent situations in which the information being exchanged is both time-sensitive and prone to rapid changes. The need to improve verbal communication in critical care medicine and specifically within perinatal care is well recognized, and has led to adaptations of one of the most commonly used communication tools (SBAR, Situation, Background, Assessment, and Recommendation) and numerous iterations of team training programs such as TeamSTEPPS [49,52,53,54,55,56,57,58]. A standardized communication lexicon specific for neonatal resuscitation has also been developed to facilitate the exchange of information among neonatal resuscitation team members and the timely implementation of the appropriate resuscitative measures [59]. However, all of these solutions require additional training and are therefore relatively rudimentary from a macroergonomics perspective. Seeking to improve the design of the system, rather than just focusing on training users, creates opportunities to find new solutions and design for human fallibilities in order to produce a stronger system. The supplementation of training methodologies with other systemic changes that support the transmission of vital information and limit the possibilities for human error will require the study of systemic weaknesses and innovative solutions such as the creation of a visual display tailored to address common lapses in information exchanged [47].

## 4. Cognitive Ergonomic Challenges

The field of cognitive ergonomics specifically focuses on the mental processes required to perceive and comprehend an environment. Understanding the factors that influence the cognitive functioning of HCPs is a critical component of safe and effective neonatal resuscitation, especially because the number of devices and displays available for monitoring neonates during the transitional period immediately after birth has risen significantly since the first publication of the review of the science underlying neonatal resuscitation by the International Liaison Committee on Resuscitation (ILCOR) in 2005 [60]. In the most recent summary of clinical guidelines published in 2015, the American Heart Association supported the use of pulse oximetry and ECG electrodes, and acknowledged the potential role of exhaled carbon dioxide monitors during neonatal resuscitation [2]. While these devices are standard of care for patients receiving respiratory support outside of the delivery room, their use during resuscitation presents unique challenges. These challenges include but are not limited to the need for: rapid application to the patient (within seconds); secure adherence even during procedures such as chest compressions; avoidance of interference with interventions such as emergent umbilical vein cannulation; interpretation of multiple data streams that require translation into actionable information; and the avoidance of confusion and non-response (“alarm fatigue”) to data that deviate out of the normal range. Hence, all of these technologies compete for the attention of HCPs who must simultaneously and continuously assess and interpret the clinical signs displayed by the patient. Every 30 s, the NRP algorithm has an evaluation or decision point that can lead to additional interventions (e.g., positive-pressure ventilation (PPV), intubation, chest compressions, umbilical venous catheter placement, or medication administration) [5]. In a typical delivery room setting, the primary responders consist of only one or two HCPs. In the event of a critically ill neonate, the number and complexity of tasks and cognitive demands on the team rapidly increase, correlating with an increase in overall errors and deviations from the NRP algorithm [61].

### 4.1. Utilizing Technological Solutions to Address Cognitive Ergonomic Challenges

With the combination of time pressure and limited number of HCPs available for immediate resuscitation, it is critical to evaluate the cognitive load generated by additional monitoring technologies and their effect on human performance. Humans have an inherently limited capacity to process data and translate it into actionable information; therefore, all technologies have the potential to aid or distract the efforts of HCPs. As new technologies are introduced into the resuscitation environment, attention to cognitive ergonomics can serve to balance the benefits and risks of those data streams in order to optimize their cognitive load on HCPs, improve human performance, and limit error.

Decision support tools have begun to enter the resuscitation environment in an effort to address this need [62,63]. The effect of a tablet-based decision support tool on human performance has been examined during standardized simulated neonatal resuscitations [64]. This device continuously displays the patient data necessary to guide neonatal resuscitation (e.g., heart rate, pulse oximetry, patient weight, and a timer) and provides a combination of visual and auditory prompts based on the NRP algorithm. The numbers displayed are color coded based on the clinical appropriateness of their values. For example, when the heart rate is greater than 100 beats per minute (bpm), it is displayed in green. For heart rates between 60 and 99 bpm, it is displayed in yellow, and a heart rate less than 60 bpm is displayed in red. Guided by the patient’s heart rate, the decision support tool provides auditory and visual recommendations for the next steps in resuscitation as indicated by the NRP algorithm. In this study, HCPs using this decision support tool exhibited significantly fewer deviations from the NRP algorithm compared to those working from memory alone, including more timely performance of PPV and chest compressions.

In a study of decision support tools in actual patient care, use of the Transitional Oxygen Targeting System (TOTS) software has been compared to the use of pulse oximetry alone [65]. The TOTS provides a graphic display of predetermined high and low pulse oximetry limits over time along with a real-time display of the patient’s current hemoglobin oxygen saturation as measured by pulse oximetry. This system provides a visual target for resuscitation teams as they titrate the oxygen to achieve the desired oxygen saturation on a minute-to-minute basis. In a study of 40 preterm infants who required supplemental oxygen in the delivery room, pulse oximetry values were maintained within the specified target range for a significantly longer period of time in neonates resuscitated using the TOTS display compared to those resuscitated using pulse oximetry alone.

Using visual and auditory mechanisms to simplify data input, decision support tools such as these reduce cognitive workload and thereby create the opportunity for improved situation awareness and human performance during neonatal resuscitation.

### 4.2. Considerations for Incorporating Additional Devices into the Resuscitation Environment

As new devices become available and are considered for incorporation into neonatal resuscitation, it is critical to ensure that they provide actionable information and their impact on human performance has been appropriately evaluated. For example, in neonatal resuscitations requiring PPV, there is increasing evidence for the inclusion of respiratory function monitors (RFMs) [66,67,68,69,70]. These devices traditionally monitor airway pressure and gas flow during PPV. Neither of these parameters are routinely available during resuscitation, but if provided, may help to optimize the delivery of PPV and ultimately improve patient outcomes. However, incorporating these additional raw data streams into a resuscitation environment that already has a native cognitive load would add significant demands for interpretation and analysis by HCPs. Technology should be leveraged to further process and translate this data into actionable information. Feedback indicating the presence of an airway obstruction or the degree of mask-leak coupled with prompts for corrective actions would function to reduce rather than increase cognitive load and could improve the performance of the team.

In addition to the provision of actionable information, devices should be evaluated for their impact on human performance. Pedi-Cap^®^ is a colorimetric carbon dioxide (CO_2_) detector that is routinely used to assess endotracheal tube (ETT) placement and confirm appropriate subsequent gas exchange by changing color from purple to yellow in the presence of CO_2_. HCPs incorporate the information from these devices and other clinical cues to assess ETT placement, but anecdotal evidence suggests that HCPs rely heavily on these devices to determine whether or not the ETT is in the trachea. Recognition of such cognitive biases is key to supporting safe patient care. In a clinical study comparing the use of the Pedi-Cap^®^ with CO_2_ flow sensors (the current gold standard for detecting exhaled CO_2_) for the determination of correct ETT placement, the PediCap^®^ failed to change color in 31% of 35 neonatal intubations, despite the flow sensor indicating correct ETT placement [71]. This failure could mislead HCPs to perform repeated and unnecessary intubations due to falsely placed confidence in the data coming from the technology intended to augment safer care.

As resuscitation recommendations evolve, more devices will enter the clinical environment and compete for the attention of HCPs. These devices should preferably provide actionable information in addition to raw data requiring recognition, interpretation, and decision-making. Human factors testing to evaluate how the device, associated data streams, and additional auditory or visual cues impact the human performance of the team should be conducted in a highly controlled simulated environment prior to use on actual patients.

## 5. Conclusions

While the research and scientific findings related to neonatal resuscitation are aptly focused on optimizing the physiological support provided to the newborn in distress, the evolving technology and work environments in healthcare will require the application of human factors and ergonomics science in order to optimize the performance of the humans caring for those patients. The application of human factors and ergonomics principles is currently a little-studied area in neonatal resuscitation. An understanding of physical, organizational, and cognitive ergonomics principles can facilitate the development of technology, devices, systems, and work environments that will support human performance, minimize the risk of error, and ensure safer and more effective patient care.
